# Zirconia based superhydrophobic coatings on cotton fabrics exhibiting excellent durability for versatile use

**DOI:** 10.1038/srep18503

**Published:** 2015-12-18

**Authors:** Indranee Das, Goutam De

**Affiliations:** 1 Nano-Structured Materials Division, CSIR-Central Glass and Ceramic Research Institute, Kolkata 700032, India

## Abstract

A fluorinated silyl functionalized zirconia was synthesized by the sol-gel method to fabricate an extremely durable superhydrophobic coating on cotton fabrics by simple immersion technique. The fabric surfaces firmly attached with the coating material through covalent bonding, possessed superhydrophobicity with high water contact angle ≈163 ± 1°, low hysteresis ≈3.5° and superoleophilicity. The coated fabrics were effective to separate oil/water mixture with a considerably high separation efficiency of 98.8 wt% through ordinary filtering. Presence of highly stable (chemically and mechanically) superhydrophobic zirconia bonded with cellulose makes such excellent water repelling ability of the fabrics durable under harsh environment conditions like high temperature, strong acidic or alkaline solutions, different organic solvents and mechanical forces including extensive washings. Moreover, these coated fabrics retained self-cleanable superhydrophobic property as well as high water separation efficiency even after several cycles, launderings and abrasions. Therefore, such robust superhydrophobic ZrO_2_ coated fabrics have strong potential for various industrial productions and uses.

Inspired by water-repellent properties of the lotus leaf and water strider’s leg in natural world, various artificial superhydrophobic surfaces have been developed for their huge potential applications including self-cleaning, anti-fouling, water treatment, anti-corrosive etc^1^,^2^,^3^,^4^,^5^. Different micro/nanoscale binary structured superhydrophobic surfaces with a high static water contact angle (WCA > 150°) and low hysteresis (CAH < 10°) have opened up new possibilities of applications in industrial and biological fields^6^,^7^,^8^,^9^,^10^,^11^. Such unique micro/nano structures play a crucial role for the development of bio-inspired special wettability in textile industries concerned with cloths/paper or oil (or organic solvents)-water separation as well as to resolve the problem takes place from oil spill accidents^12^,^13^. Textiles as such demonstrate intrinsic porous, rough, flexible and hydrophilic surfaces with high absorption ability, however they absorb both water and oil^14^,^15^. It is noteworthy that the textile surfaces containing larger amount of fluorinated or perfluorinated materials possess very low surface energy and show super-repellent property for all kinds of liquids^16^,^17^,^18^. Those surfaces not only repel lower surface tension liquids, such as oil, alcohols but also repel higher surface tension liquids, such as water^16^,^17^,^18^. As a result, such coated textiles are failed to perform oil (or organic solvents)-water separation and solve the troubles occur because of oil leakage. Therefore, prudent chemical modifications are needed to control the selectivity and wettability of textiles for removal of oil from water by tuning the micro/nanoscale roughness and surface energy^14^. Recently, textile surfaces with superhydrophobic and superoleophilic properties have attracted massive interest in the field of commercial oil/water separation and to prevent the environmental hazard causes by frequent oil spill mishap or industrial oily wastewater^19^,^20^. It is mainly because of their capacity of absorbing oil while repelling water completely, exhibiting high separation efficiency and selectivity^19^,^20^,^21^. According to the Cassie state which is based on the consideration of such textile’s wettability^14^, the water droplets form spheres and reside on the top of the micro/nano structured fibrous surface without filling up the nanogrooves to maintain superhydrophobicity^22^,^23^. However, such superhydrophobic materials on fabrics have certain limitations for potential applications due to the time-consuming chemical preparation, low chemical-mechanical stability, poor selectivity and reusability, and limited large scale fabrication^19^,^21^. In case of many practical applications, it is desirable for superhydrophobic materials to be stable in both acidic and basic conditions for a prolonged length of time. But it is observed that the hydrophobicity of the various composites reduces with time in presence of strong acidic or alkaline solution. In addition, the water repelling ability of silica–based hydrophobic coating decreases within certain hours in basic condition^24^,^25^ because of the dissolution of Si–O–Si network at high pH^26^.

Zirconia is well known for its strong covalent character, excellent mechanical strength with a very high bond dissociation energy (~753 kJ mole^–1^), thermal stability as well as strong alkali and acid resistant property compared to other ceramic materials^27^,^28^,^29^,^30^,^31^. On the other hand, fluorinated methyl groups are less reactive as well as possess lower surface energy compared to the normal hydrophobic –CH_3_ groups due to the presence of strong C–F bonds^32^,^33^. Keeping in view these issues, in this work we have fabricated a highly stable sol-gel derived fluorinated silyl functionalized zirconia (fsZr) on fabrics by simple immersion technique without deteriorating the original flexibility and color of the fabric. During the preparation of coating sol, a very minor fraction of fluorinated triethoxysilane (FDTES) was added into the zirconia sol and co-hydrolyzed to make covalent bonding through Zr–O–Si linkages. After immersion of fabric into such functionalized sol, the free hydroxyl groups of zirconia easily interacted with the hydrophilic sites associated with cellulose. Thus strong bonding between fabric and functionalized zirconia was formed by condensation after systematic thermal treatment. It is noteworthy here that unlike the silica based and other hydrophobic coatings which were not stable enough for day-to-day practical uses, the fsZr coating on fabrics was found to be extremely sustainable and durable. This is because zirconia is strong resistant to acid, alkali, organics and mechanical as well as thermal stresses. As a result, the fsZr coating remains efficient to preserve the superhydrophobic property as well as high water separation efficiency even under severe environment for longer period of time. Therefore, this robust and self-cleanable fabric expected be used not only in repeated oil/water separation but also to manufacture superhydrophobic military suits, garments for different labs, daily uses etc. To the best of our knowledge such zirconia based superhydrophobic cum superoleophilic cotton fabric with extraordinary stability and versatility has not yet been reported. The fabricated reinforced superhydrophobic coating on fabric was characterized by FTIR, TGA, FESEM, TEM and XRD. The abrasion resistance, washing durability, water repellence and self-cleaning ability of the coated fabrics were investigated by rubbing with sand paper with load, several cycles of launderings and water contact angle studies under different conditions, respectively. The chemical stability of the fabrics was examined through the immersion into strong acidic and alkaline solutions, and different organic solvents.

## Results

### The principle of superhydrophobic fabric preparation

**Figure 1:**
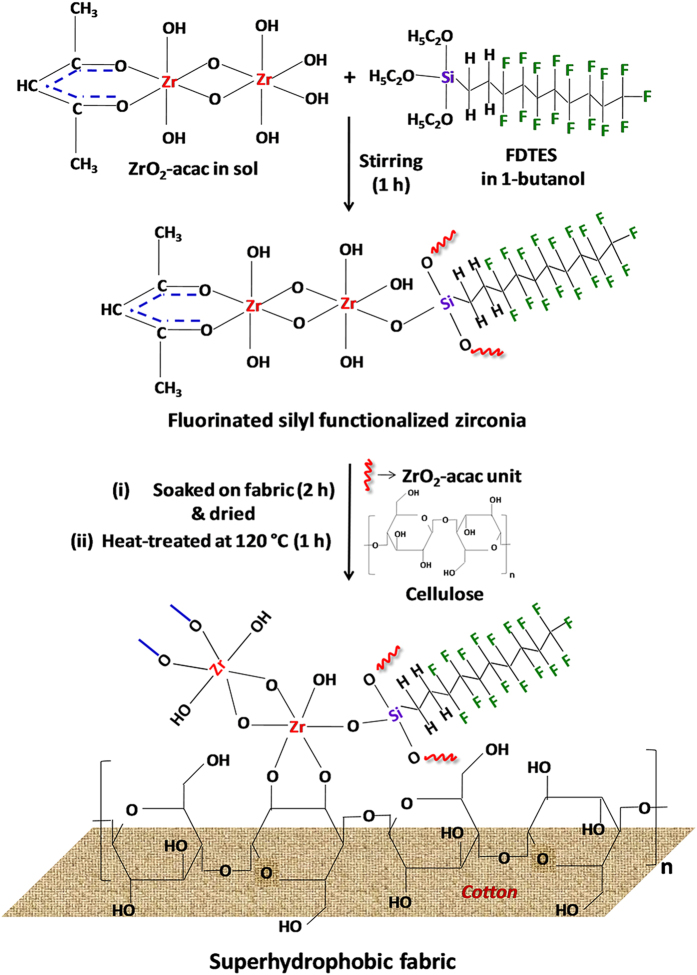
Schematic representation of fsZr coated fabric. The reaction principle shows the formation of fluorinated silyl functionalized superhydrophobic zirconia coating on cotton fabric.

The fluorinated silyl (fs) functionalized zirconia sol (designated as ‘fsZr’ sol) formation along with superhydrophobic/superoleophilic fabric production is shown in Fig. 1. The original zirconium (IV) n-propoxide (ZP) exists in dimeric form by coordinating alcohol molecule. After adding acetylacetone (ZP:acac = 2:1), dimeric alkoxide transformed into partial acetylacetonate chelate after substitution, and hydrolyzed under mild-acidic condition (henceforth designated as ‘acZr’ sol)^34^,^35^. Such acac modification is necessary to control the very fast hydrolysis rate of ZP^35^. It is expected that after mixing of FDTES solution with the acZr sol (keeping equivalent ZrO_2_:fs molar ratio 1:0.033), some OH groups attached with zirconia units condensed with the hydrolyzed fluorinated silane (as shown in the scheme of Fig. 1). When the fabric was immersed in the above sol, plenty of free OH groups associated with zirconia were condensed with the OH groups of the cellulose unit, and after thermal treatment robust superhydrophobic fabric was formed by the elimination of water molecules. It should be noted that the increasing of immersion time (more than 2 h) and molar concentration of equivalent ZrO_2_ in the sol, reduce the original softness, color and flexibility of the cotton fabrics. Therefore, the immersion time and concentration of the components in sol were optimized to obtain this highly efficient, flexible and durable superhydrophobic/superoleophilic cotton fabric for versatile uses.

### Chemical state and surface structure

**Figure 2:**
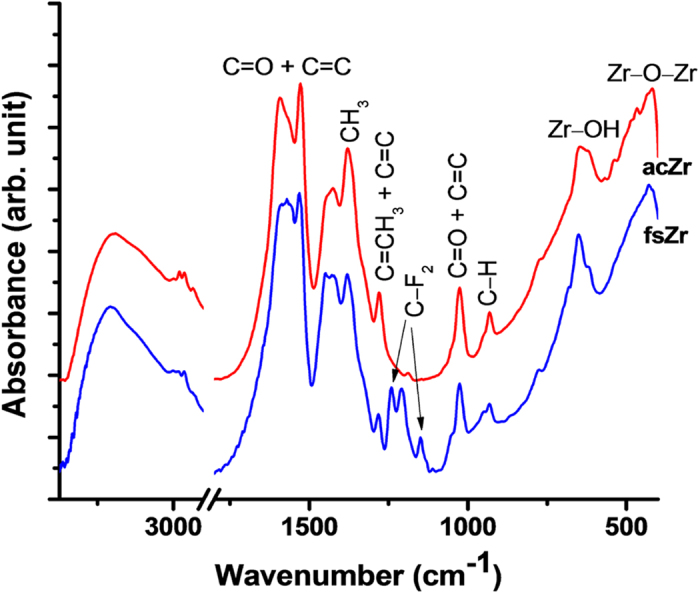
FTIR spectra of the samples heat-treated at 120 °C for 1 h. FTIR spectra indicates the presence of –CF_2_ groups in fsZr coating material which is absent in case of control acZr powder. Both the samples show zirconia related peaks.

FTIR studies of the control acZr powder and fsZr coating material heat-treated at 120 °C were undertaken to understand the chemical bonding (Fig. 2). The bands at 1602, 1525 and 1384 cm^–1^ can be assigned due to the acac chelates^34^. A slight decrease of these acac chelates related peak intensities in case of fsZr compared to control acZr can be due to the partial decomposition of acac chelates during hydrolysis, and subsequent heat-treatment which is also observed from the thermogravimetric analysis (TGA) (Supplementary Fig. S1). The peaks at 650 and 430 cm^–1^ can be attributed for the vibration of Zr–OH and Zr–O–Zr linkages, respectively^36^,^37^. New peaks in case of fsZr near 1240–1150 cm^–1^ region were originated from the –CF_2_ groups present in fluorinated silyl functions attached with the zirconia^38^. The band centered at 3431 cm^–1^ is due to the OH stretching present in the materials. It can be noted that the FTIR spectra of as prepared and heat-treated (200 and 300 °C for 1 h) fsZr coating materials showed no noticeable changes in the characteristic peak intensities of –CF_2_ groups (Supplementary Fig. S2) indicating thermal stability of CF_2_ groups up to 300 °C.

**Figure 3:**
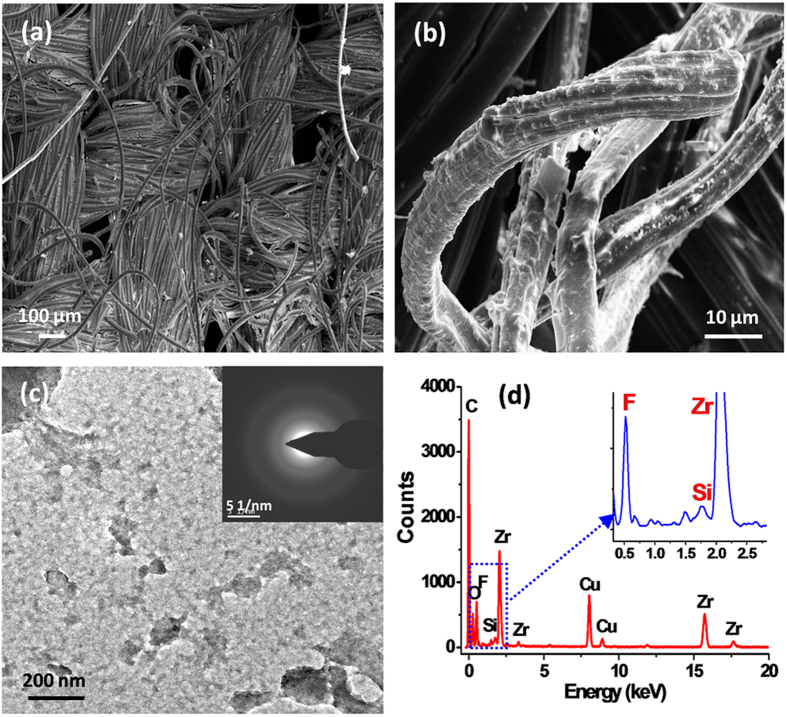
FESEM and TEM analyses of the fsZr coating heat-treated at 120 °C. (**a**) FESEM image of fsZr coated cotton fabric and (**b**) the corresponding higher magnification image showing the surface microstructure of the coating. (**c**) TEM bright field image showing some fragments of coating. Inset in (**c**) represents the SAED pattern of fsZr. (**d**) EDX acquired from the bright field image shows the presence of Zr, F and a very small amount of Si in the coating. A magnified EDX spectrum (0.3–2.8 keV) is given in the inset of (**d**) to show the peaks for F–K and Si–K lines.

Surface morphology of the substrate plays a vital role in superhydrophobicity. Therefore, the surface topography of the fsZr coated cotton fabric cured at 120 °C was observed by FESEM (Fig. 3). Figure 3a shows the presence of rough surface topography on fabrics with clusters of coating material in the gap between the fibres. The magnified FESEM image of the coated fabric (Fig. 3b) reveals the microstructure of the surface with homogeneously distributed coating materials. Whereas a tightly woven and smooth structure is clearly observed in the uncoated original fabric (Supplementary Fig. S3).

The nature and composition of the zirconia based coating material were investigated by transmission electron microscopic (TEM) studies (as described in the experimental section). The bright field TEM image shows some fragments of the coating material from the fabrics (Fig. 3c). The SAED pattern obtained from those parts reveals the amorphous nature of the fsZr (inset in Fig. 3c) which is also in agreement with the wide angle XRD pattern of the fsZr coating material (Supplementary Fig. S4). EDX pattern (Fig. 3d) acquired from the different portions of the material confirms the presence of Zr, F and a very small amount of Si elements in the coating. Signals of Cu observed in the EDX are from Cu grid used for the TEM study. A semi-quantitative analysis from the Zr–K and Si–K (originated from fluorinated silyl group) lines of EDX reveals the molar ratio of Zr:Si = 1:0.037 (average of 3 sets of data).

### Evaluation of superhydrophobicity and self-cleaning ability

**Figure 4:**
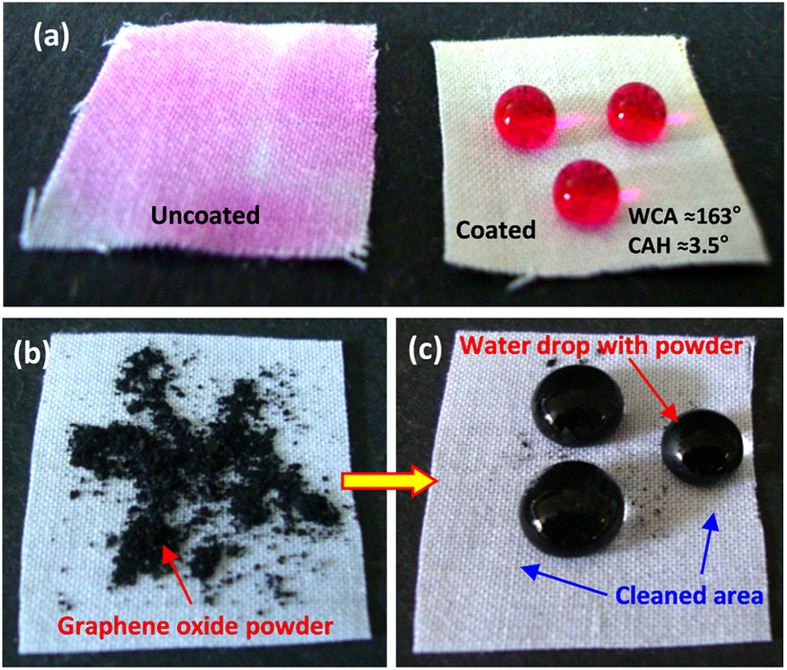
Photographs of the uncoated and fsZr coated fabrics. (**a**) The dyed (Rhodamine B) water drops on uncoated and fsZr coated cotton fabrics. (**b**) Graphene oxide powder on the coated superhydrophobic fabric. (**c**) Water drops with graphene oxide powder showing the self-cleaning ability of the fsZr coated fabric.

The fluorinated silyl functionalized zirconia coated fabrics (Fig. 4) revealed superhydrophobicity with high water contact angle (WCA) ≈163 ± 1° and low contact angle hysteresis (CAH) ≈3.5°. The coated fabric did not undergo wetting and dyed water drops formed spheres on it while the uncoated fabric became complete wet, and was stained by dyed water drops instantly (Fig. 4a). When we poured tea drops on this fsZr coated cotton fabric, the tea drops also formed complete spheres on it without staining the surface (Supplementary Fig. S5a). Similarly when hydrophilic graphene oxide (GO) powder was placed on the fsZr coated fabric (Fig. 4b), and followed by pouring a little water, spherical drops were formed immediately with GO powder turning the fabric dirt free (Fig. 4c).The above observations clearly support the excellent water repelling, stain-resistant and self-cleaning abilities of the fsZr coating. The coated superhydrophobic fabric can also float freely on the water surface (Supplementary Fig. S5b) whereas upon forceful immersion into water, mirror like appearance on the fabric surface can be observed (Supplementary Fig. S5c) and fabric remained completely dried after taking out from water. On the other hand, the coated fabric soaked hexane and instantly went under the beaker representing its superoleophilic property (Supplementary Fig. S5d).

### Thermal stability of the coating

To study the thermal stability of fluorinated silyl modified superhydrophobic zirconia coatings on fabrics with respect to the WCA and CAH, the coated samples were heat-treated in air. We found that heat-treatment of coated fabric at 200 °C for 2 h maintained its color and flexibility like the original uncoated one with superhydrophobicity (Supplementary Fig. S6a,b). We observed yellowing of the coated fabric on prolonged storing at 200 °C ( > 2–12 h) due to the deterioration of cellulose component of cotton^39^. Such prolonged thermal treatment however does not affect the superhydrophobic characteristics (Supplementary Fig. S6c) of the coating because of the thermal stability of –CF_2_ groups.

### Chemical stability

**Figure 5:**
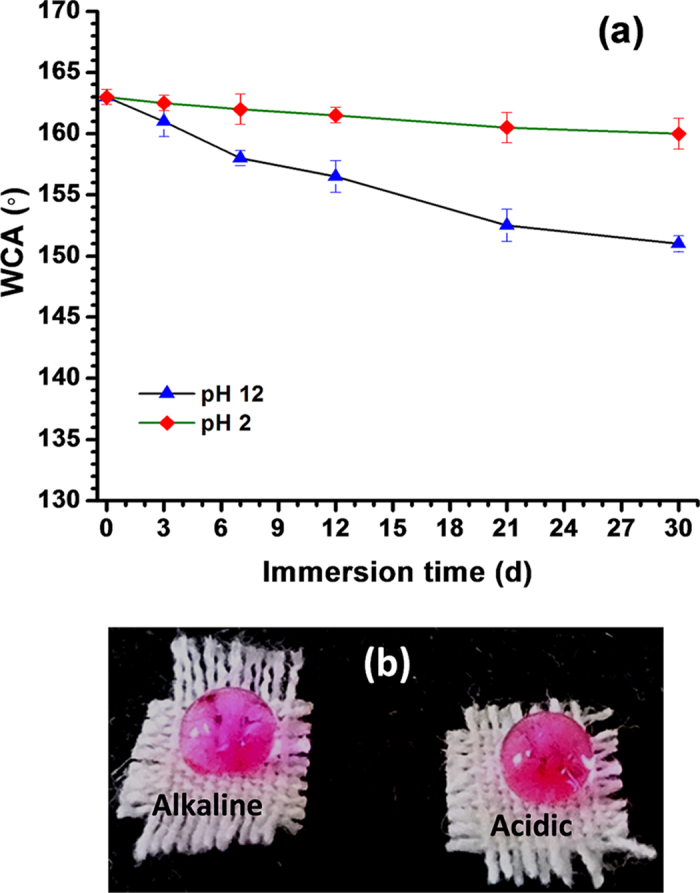
Plot and photograph showing the acid and alkali resistant property. (**a**) Plots revealing the changes in water contact angle of superhydrophobic fabrics during immersion into strong acidic and alkaline solutions up to 30 d. (**b**) Photographs showing the dyed water drops on coated fabrics after 30 d of immersion in strong acidic and alkaline solutions.

Various superhydrophobic materials lose their hydrophobicity in presence of strong acid, alkali and different organic solvents within certain hours. Considering these crucial issues, we investigated the chemical durability of this fluorinated silyl modified zirconia based superhydrophobic coating by measuring the variations of WCA and CAH during immersion of the fabric into strong acidic (pH = 2) and alkaline (pH = 12) solutions (Fig. 5)^14^,^24^,^25^. Interestingly the WCA of the coated fabric was almost unchanged (≈160 ± 2°) and CAH was close to 4.6° after 30 d of immersion in strong acidic solution whereas in case of strong alkaline solution, slight change in WCA ≈151 ± 1° and CAH ≈8.5° were observed (as shown in Fig. 5a) after such long immersion. In addition, the water drops formed complete spheres on the superhydrophobic fabrics even after this long period (30 d) of immersion into both the solutions (Fig. 5b).

To check further chemical stability of the superhydrophobic coating, the coated fabrics were immersed in various organic solvents^25^ for longer period of time. For all the five organic solvents tested, the water contact angles showed no change within experimental error over 14 d of immersion (Supplementary Table S1).

### Application in oil/water separation

**Figure 6:**
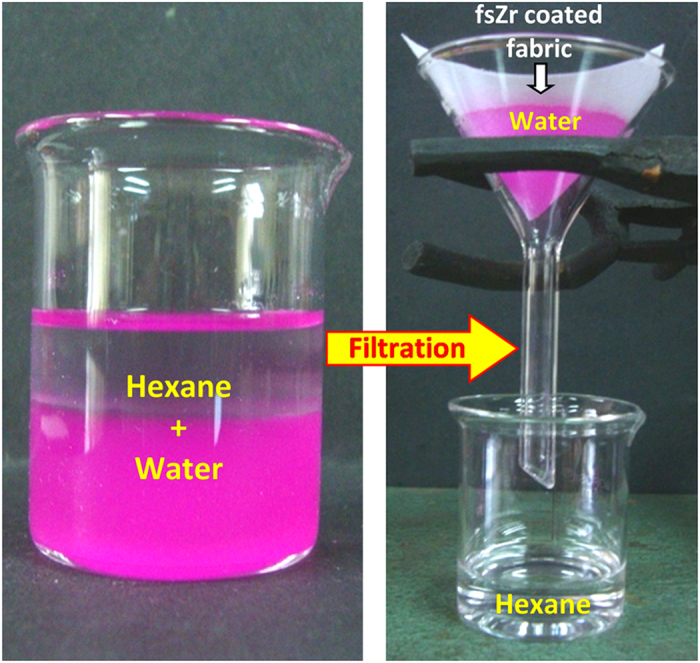
Image showing the separation process for hexane-water mixture. Hexane freely permeated and rapidly accumulated into the bottom of the beaker through the fsZr coated superhydrophobic/superoleophilic cotton fabric whereas water (dyed with Rhodamine B) still remained on the textile surface.

Since the fluorinated silyl functionalized zirconia on fabrics showed excellent water repelling and superoleophilic property with chemical stability, we performed the water separation experiments on coated “filter cloth” as shown in Fig. 6. After mixing vigorously, when hexane-water mixture was poured onto the superhydrophobic cotton fabric, hexane immediately spread and freely permeated through the fabric at atmospheric pressure and, rapidly accumulated into the bottom of the beaker. It can be clearly seen in Fig. 6 that there was no water in the separated hexane. On the other hand, water (red colored by Rhodamine B) still remained on the textile surface. The water separation efficiency of the filter fabric was defined as the weight ratio of the collected water after filtration to the initially added water^40^. The prepared fabric could be repeatedly used for the rapid separation of a high oil ratio from oil/water mixtures with a high water separation efficiency of 98.8 wt% and showed appreciable superhydrophobicity with WCA ≈162 ± 1° and CAH ≈3.8° even after 10 cycles of applications (Supplementary Table S2). A coated fabric having dimension of 5 × 5 cm^2^ can separate 20 ml of oil/water mixture within 4 s by simple filtering method without applying any external force (as shown in Fig. 6).

### Mechanical durability of fsZr coating

**Figure 7:**
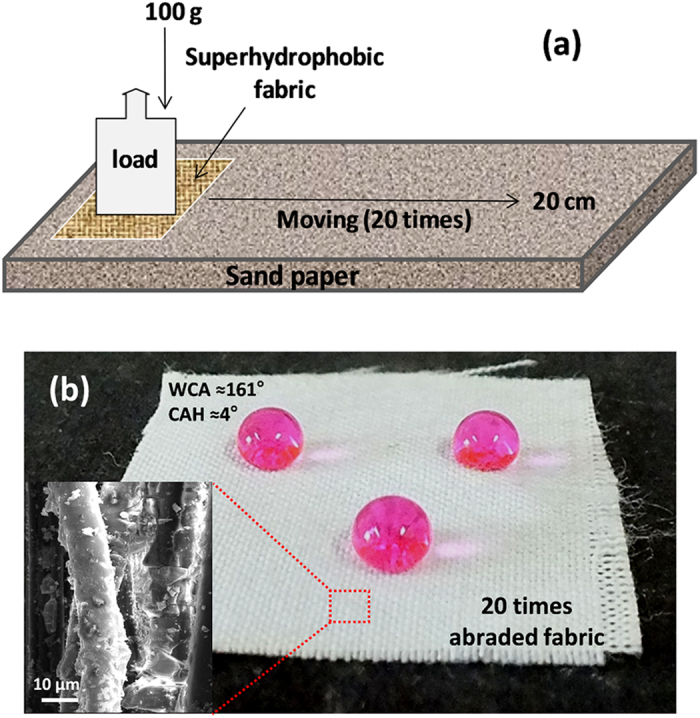
Mechanical stability of fsZr coating. (**a**) Schematic illustration of the abrasion test of the coated fabric on sand paper. (**b**) The image of dyed water drops on the abraded (20 cycles) coating surface of cotton fabric. Inset in (**b**) reveals insignificant damage of the coated fabric even after 20 times of abrasion test.

The robustness of the coating on cotton cloth was evaluated by the abrasion test using 80 mesh sand paper as a quite harsh abrasion partner^40^,^41^,^42^,^43^ (see schematic illustration in Fig. 7a) as described in the experimental section. Before and after 20 cycles of abrasions following the similar procedure, the change in WCA of the abraded surface was measured. It was found that even after 20 times of abrasion there was no noticeable change in the water contact angle as well as the surface topography of the fabric (inset in Fig. 7b). Even after this abrasion test, the oil/water separation efficiency of fabric remained above 97.7% and the water drops formed spheres on it with WCA ≈161.5 ± 1° and CAH ≈4° by maintaining the non-wetting property like original coated fabric (Fig. 7b). Moreover, mechanical durability of this superhydrophobic coating was also tested through the adhesion of single sided tape and twisting by hand (Supplementary Fig. S7a,b)^41^. The WCA and CAH remained identical with the newly coated surface after these qualitative mechanical stability tests.

**Figure 8:**
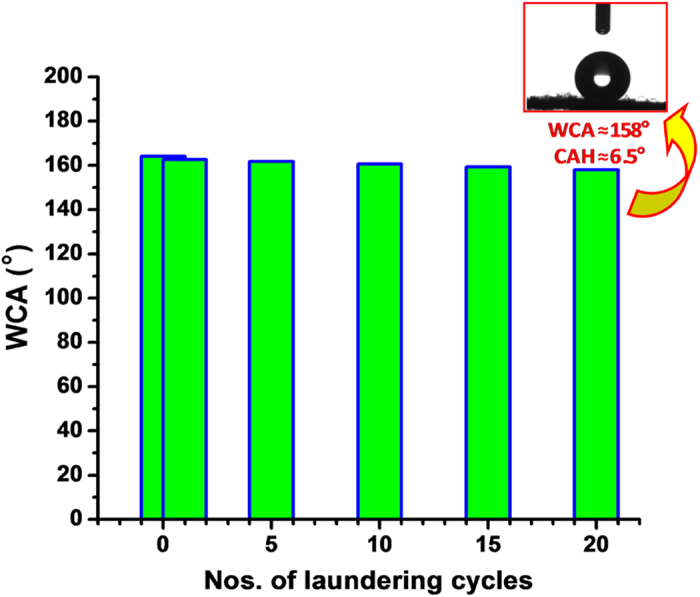
Washing durability of the fsZr coating. Plot showing the water contact angle values of coated superhydrophobic fabric after different number of washing cycles following standard AATCC test method 61–2006 (test no. 2 A). One cycle under this specification is equivalent to 5 home laundering by washing machine. Inset shows the image of water drop on the coated fabric after 20 cycles of this standard laundering test.

Water contact angles on the fsZr coated cotton fabrics measured up to 20 simulated washing cycles are presented in Fig. 8. Water repelling ability of the washed samples remained intact with a very minor alteration of WCA and CAH up to 20 cycles of laundering following standard AATCC test method (equivalent to 100 cycles of home laundering). The coated fabrics showed more than 97% oil/water separation efficiency even after 20 cycles of this machine laundering.

## Discussion

A fluorinated silyl functionalized zirconia (fsZr) based superhydrophobic and superoleophilic coating was fabricated on cotton fabric. The FTIR spectral analysis (Fig. 2) of the coating material reveals the existence of signature peaks of zirconia along with the characteristic peaks originated from –CF_2_ groups present in fluorinated silyl functions. The molar ratio of Zr:Si in fsZr coating obtained from semi-quantitative analysis using TEM-EDX (Fig. 3d) is consistent with the nominal composition used for sol preparation. The coated cotton fabric shows excellent stain-resistant and self-cleaning abilities (Fig. 4) along with superoleophilic property (Supplementary Fig. S5d) due to the combined effect of C–F groups possessing very low surface energy in addition with the micro level rough fibrous exterior surface. The superhydrophobic character of this fsZr coated fabric surface can be explained by the Cassie-Baxter model where the water droplets form spheres and reside on the top of such durable dense rough surface (as observed in Fig. 3a,b) but do not fill up the nanogrooves^44^. The important criteria for the practical uses of the superhydrophobic textiles are their durability and reusability. However, it is observed that the various superhydrophobic materials (silica and other) lose their hydrophobicity in presence of strong acid, alkali and different organic solvents within certain hours. This drop of water repellency could happen due to the instability of the components present in those superhydrophobic materials in strong acidic and alkaline medium, and the non-polar–non-polar interactions in between the materials and organic solvents^12^,^24^. Whereas this fsZr coating showed outstanding chemical stability after immersion in strong acidic and alkaline solutions (Fig. 5), and different organic solvents (Supplementary Table S1) for a longer period of time. The persistence of superhydtophobicity of the coated cotton fabric even after immersion into strong alkaline medium up to one month was not shown by any research group. In this case, such excellent chemical stability and reusability of the coatings were achieved mainly due to the use of zirconia based (fsZr) coating which is chemically inert as well as strong alkali-acid resistant^28^,^29^. More elaborately, due to the highest bond dissociation energy and strong covalent nature of Zr–O–Zr compared to Si–O–Si, Ti–O–Ti, Al–O–Al etc, the hydrolysis and dissolution of that network is prohibited at very low and high pH, respectively^26^ which facilitate the chemical stability of fsZr coating. Further, the less reactive fluorinated silyl functional groups having lower surface energy than other non-polar components successfully restrict the contact of acidic/alkaline aqueous solutions to the Zr–O–Si linkage side in coating material^32^,^33^. As a result, the whole coating remained firmly bonded with the cellulose units even under a very harsh chemical environment. The difference between water and oil on this type of surface with special wettability results in one intrinsic application in oil/water separation. Moreover, the textiles could be a better candidate due to their soft and flexible nature. Since the fluorinated silyl functionalized zirconia on fabrics showed excellent water repelling as well as superoleophilic property with enormous chemical stability, we performed the water separation experiments on coated “filter cloth” (Fig. 6). This coated fabric exhibits considerably high water separation efficiency without deteriorating its original WCA and CAH even after several cycles of separations. To the best of our knowledge, few successful superhydrophobic fabrics for oil/water separation have been reported due to the instability of superhydrophobic material in non-polar solvents^12^,^24^. The excellent stability and reusability of the fsZr coated fabrics under different conditions could provide more opportunities for numerous practical applications. Similarly, this coated fabric retained its superhydrophobicity and high oil/water separation efficiency also after several cycles of sand paper abrasion test (Fig. 7) and standard launderings (Fig. 8). It is noteworthy here that such excellent abrasion and laundering durability as well as reusability of the coating can also be described as the combination of mechanical robustness and chemical inertness of the fabricated zirconia based superhydrophobic coating covalently bonded with cellulose of the fabric. Besides repeated oil/water separations, such washing durability also recommends the utility of these coated fabrics in superhydrophobic garment manufacturing for military, different labs and daily uses.

In conclusion, we have demonstrated a simple, new and innovative approach to fabricate an exceptionally stable zirconia based superhydrophobic as well as superoleophilic coating on cotton fabric. The existence of chemically inert and mechanically durable fluorinated silyl functionalized zirconia bonded with cellulose makes the superhydrophobicity of coating material sustainable under severe environment conditions such as high temperature, corrosive solutions, various organic solvents, and mechanical forces for longer period of time. The coated fabric possesses high water separation efficiency even after several cycles of treatment in different conditions. Undoubtly, zirconia present in this fsZr coating plays the key role for generation of such extraordinary stability for day-to-day practical uses which cannot be achievable by any other superhydrophobic composite. Due to these outstanding mechanical and chemical robustness as well as self-cleanable features, these fsZr coated superhydrophobic fabrics could be employed to manufacture oil/water separation apparatus, military suits, lab coats, medical clothing and daily garments. Thus this newly designed fsZr coating has immense potential to being revolution in the field of technical textiles with various functionalities for the benefit of humanity.

## Methods

### Materials

Zirconium (IV) n-propoxide (ZP) in n-propanol (70%) and 1 H, 1 H, 2 H, 2 H-perfluorodecyl triethoxy silane (FDTES) (97%) were purchased from Alfa-aesar. 1-Propanol and nitric acid were purchased from RANKEM. 1-Butanol and acetylacetone (acac) were bought from Merck. White commercial cotton fabric was obtained from a local fabric store and rinsed with ethanol, and distilled water before use followed by washing exhaustively with an excess of water and drying at 50 °C for 30 min^24^.

### Preparation of fluorinated silyl functionalized zirconia (fsZr) solution

At first 9.4 g ZP was dissolved in 9.4 g 1-butanol in a beaker. Simultaneously, in another 100 ml beaker 1.4 g acetylacetone and 9 g 1-butanol were taken. The ZP solution was then added into the acetylacetone solution with stirring, and the stirring was continued for 1 h for complete chelation to stabilize the ZP. The molar ratio of ZP:acac was 2:1. After that 1 g distilled water, 5 g 1-propanol and 0.02 g 1(N) HNO_3_ were added and mixed well with the stabilized ZP solution by constant stirring. At last 5 g 1-propanol and 10 g 1-butanol mixture was added to the solution, and stirred at room temperature for 1 h to prepare 5 equivalent wt% zirconia sol (acZr). The whole solution was kept overnight in the refrigerator prior to use.

In another beaker 0.2 g FDTES was dissolved in 20 g 1-butanol and stirred for 30 min. This FDTES solution was then added to the 25 g acZr sol and stirred for 1 h for homogeneous mixing to obtain the final fsZr sol. In this fsZr sol molar ratio of equivalent ZrO_2_ and fluorinated silyl (fs) group was maintained 1:0.033.

### Preparation of superhydrophobic fabric

To prepare the superhydrophobic fabric, cleaned and dried fabrics were immersed in the above homogenized fsZr solution for 2 h, dried at 60 °C for 30 min, and then cured at 120 °C for 1 h. Simultaneously, a small amount of homogenized fsZr and acZr sols were casted separately as a thin layer into different petri dishes and heat-treated in a similar manner like the coating. Thus the fsZr coating material and acZr powder (as control) were obtained, and used for characterization purposes.

### Characterizations

Fourier transformed infrared (FTIR) spectra of the acZr powder and fsZr coating material were recorded (KBr pellet method) using a Nicolet 380 FTIR spectrometer with 200 scans for each samples. TG measurement of the fsZr coating material was done using Netzsch TG209F3 Tarsus thermal analyser with a heating rate of 2 °C min^‒1^ in static air. Field emission scanning electron microscopic (FESEM) analyses of the coated and uncoated fabrics were done by Carl Zeiss, Germany, SUPRA-35VP instrument. Transmission electron microscopic (TEM) measurements were carried out with Technai G2-30ST (FEI) operating at 300 kV attached with EDX facility. For TEM analysis, a very small amount of coated fabric sample was grinded and dispersed in ethanol, and then deposited on the carbon-coated Cu grid. Three sets semi-quantitative analyses were carried out using TEM-EDX data acquired from different fragments of coating material and the average value for Zr:Si molar ratio was reported. GIXRD pattern of the synthesized fsZr coating material was recorded using a Rigaku SmartLab X-ray diffractometer operating at 9 kW (200 mA; 45 kV) using Cu Kα (λ = 1.54059 Å) radiation in powder mode at a scan rate 2° 2θ s^−1^. Static water contact angle of the coated superhydrophobic fabrics was measured with a dataphysics instrument using 4 μl water drop. The hysteresis characteristic was obtained by tilting cradle method using 20 μl water droplet under ambient condition. According to this method, the droplet is placed on the substrate which is then gradually tilted. The hysteresis is considered as the difference between advancing and receding contact angles at the same time point just before the droplet starts to move. The water contact angle data were measured in 10 different areas and average values of the data were reported. Chemical stability of the superhydrophobic/superoleophilic coating was evaluated through the immersion into strong acidic and alkaline solutions (pH = 2 and pH = 12)^14^,^24^,^25^ and five organic solvents (hexane, dimethyl formamide, ethylacetate, ethanol and acetone)^25^ for 30 and 14 d, respectively. Water separation efficiency of the coated fabrics was investigated by separating water from hexane-water mixture^12^,^40^,^45^ for 10 cycles and the change in WCA and CAH were noted before and after the test. Prior to check the WCA and CAH, the samples were dried at 60 °C for 30 min after each test. Abrasion resistant ability of the superhydrophobic fabric was performed following the sand paper abrasion test^40^,^41^,^42^,^43^. In order to check the surface robustness of the coating on fabric, the coated fabric of dimension 5 × 5 cm^2^ was dragged facing the 80 mesh sand paper surface (as a quite harsh abrasion partner) along one direction (about 20 cm) after putting 100 g load on the top of the sample. The test was repeated 20 times for the same coated sample. The change in water repellency of the surface of the coating before and after 20 abrasion cycles was examined by water contact angle measurements. Washing durability test of the coated fabrics was conducted according to the standard method for fabric coating (AATCC Test Method 61-2006, test no. 2 A). This accelerated wash procedure is equivalent to 5 cycles of home machine washing.

## Additional Information

**How to cite this article**: Das, I. and De, G. Zirconia based superhydrophobic coatings on cotton fabrics exhibiting excellent durability for versatile use. *Sci. Rep.* **5**, 18503; doi: 10.1038/srep18503 (2015).

## Supplementary Material

Supplementary Information

## References

[b1] Goswami D., Medda S. K. & De G. Superhydrophobic films on glass surface derived from trimethylsilanized silica gel nanoparticles. ACS Appl. Mater. Interfaces 3, 3440–3447 (2011). 21823656 10.1021/am200666m

[b2] Zhang Y.-L., Xia H., Kim E. & Sun H.-B. Recent developments in superhydrophobic surfaces with unique structural and functional properties. Soft Matter. 8, 11217–11231 (2012).

[b3] Sahoo B. N. & Kandasubramanian B. Recent progress in fabrication and characterization of hierarchical biomimetic superhydrophobic structures. RSC Adv. 4, 22053–22093 (2014).

[b4] D’Acunzi M. *et al.* Superhydrophobic surfaces by hybrid raspberry-like particles. Faraday Discuss. 146, 35–48 (2010). 21043412 10.1039/b925676h

[b5] Xue C.-H. & Ma J.-Z. Long-lived superhydrophobic surfaces. J. Mater. Chem. A 1, 4146–4161 (2013).

[b6] Wang H. *et al.* Preparation of lotus-like hierarchical microstructures on zinc substrate and study of its wettability. RSC Adv. 4, 33730–33738 (2014).

[b7] Milionis A. *et al.* Spatially controlled surface energy traps on superhydrophobic surfaces. ACS Appl. Mater. Interfaces 6, 1036–1043 (2014). 24386959 10.1021/am404565a

[b8] Facio D. S. & Mosquera M. J. Simple strategy for producing superhydrophobic nanocomposite coatings *in situ* on a building substrate. ACS Appl. Mater. Interfaces 5, 7517–7526 (2013). 23855260 10.1021/am401826g

[b9] Verho T., Bower C., Andrew P., Franssila S. & Ikkala O. Mechanically durable superhydrophobic surfaces. Adv. Mater. 23, 673–678 (2011). 21274919 10.1002/adma.201003129

[b10] Tarwal N. L. *et al.* Spray deposited superhydrophobic ZnO coatings via seed assisted growth. Surf. Coat. Technol. 206, 1336–1341 (2011).

[b11] Wu J. *et al.* Self-healing of the superhydrophobicity by ironing for the abrasion durable superhydrophobic cotton fabrics. Sci. Rep. 3, 2951 (2013). 24135813 10.1038/srep02951 PMC3798042

[b12] Li J. *et al.* Stable superhydrophobic coatings from thiol-ligand nanocrystals and their application in oil/water separation. J. Mater. Chem. 22, 9774–9781 (2012).

[b13] Liu X., Ge L., Li W., Wang X. & Li F. Layered double hydroxide functionalized textile for effective oil/water separation and selective oil adsorption. ACS Appl. Mater. Interfaces 7, 791–800 (2015). 25490110 10.1021/am507238y

[b14] Cortese B., Caschera D., Federici F., Ingo G. M. & Gigli G. Superhydrophobic fabrics for oil-water separation through a diamond like carbon (DLC) coating. J. Mater. Chem. A 2, 6781–6789 (2014).

[b15] Zhou H., Wang H., Niu H. & Lin T. Superphobicity/philicity janus fabrics with switchable, spontaneous, directional transport ability to water and oil fluids. Sci. Rep. 3, 2964 (2013). 24129357 10.1038/srep02964 PMC3797433

[b16] Kota A. K., Kwon G. & Tuteja A. The design and applications of superomniphobic surfaces. NPG Asia Mater. 6, (2014) e109, 10.1038/am.2014.34.

[b17] Wang H. *et al.* Durable, self-healing superhydrophobic and superoleophobic surfaces from fluorinated-decyl polyhedral oligomeric silsesquioxane and hydrolyzed fluorinated alkyl silane. Angew. Chem. Int. Ed. 50, 11433–11436 (2011). 10.1002/anie.201105069 21990122

[b18] Gagliardi D. D. & Greenwich E. Fluoroacid and zirconium oxyhalide compositions and materials treated therewith. US Patent, US 3372039 (1968).

[b19] Xue Z., Cao Y., Liu N., Feng L. & Jiang L. Special wettable materials for oil/water separation. J. Mater. Chem. A 2, 2445–2460 (2014).

[b20] Zhu Y., Wang D., Jiang L. & Jin J. Recent progress in developing advanced membranes for emulsified oil/water separation. NPG Asia Mater. 6, (2014) e101, 10.1038/am.2014.23.

[b21] Yu T., Xu G., Wang X., Yang J. & Hu J. Fabrication of oil-water separation filter paper by simple impregnation with fluorinated poly-acrylate emulsion. BioResources 9, 4421–4429 (2014).

[b22] Das I., Mishra M. K., Medda S. K. & De G. Durable superhydrophobic ZnO−SiO_2_ films: a new approach to enhance the abrasion resistant property of trimethylsilyl functionalized SiO_2_ nanoparticles on glass. RSC Adv. 4, 54989–54997 (2014).

[b23] Marmur A. Wetting on hydrophobic rough surfaces: to be heterogeneous or not to be ? Langmuir 19, 8343–8348 (2003).

[b24] Deng Z.-Y., Wang W., Mao L.-H., Wang C.-F. & Chen S. Versatile superhydrophobic and photocatalytic films generated from TiO_2_-SiO_2_@PDMS and their applications on fabrics. J. Mater. Chem. A 2, 4178–4184 (2014).

[b25] Zhao Y., Xu Z., Wang X. & Lin T. Photoreactive azido-containing silica nanoparticle/polycation multilayers: durable superhydrophobic coating on cotton fabrics. Langmuir 28, 6328–6335 (2012). 22462539 10.1021/la300281q

[b26] Jaroniec C. P., Jaroniec M. & Kruk M. Comparative studies of structural and surface properties of porous inorganic oxides used in liquid chromatography. J. Chromatogr. A 797, 93–102 (1998).

[b27] Ramesh T. R., Gangaiah M., Harish P. V., Krishnakumar U. & Nanandakishore B. Zirconia ceramics as a dental biomaterial-an over view. Trends Biomater. Artif. Organs 26, 154–160 (2012).

[b28] Tan D. *et al.* Synthesis of nanocrystalline cubic zirconia using femtosecond laser ablation. J. Nanopart. Res. 13, 1183–1190 (2011).

[b29] Family R., Solati-Hashjin M., Nik S. N. & Nemati A. Surface modification for titanium implants by hydroxyapatite nanocomposite. Caspian J. Intern. Med. 3, 460–465 (2012). 24009915 PMC3755845

[b30] Harada K., Shinya A., Yokoyama D. & Shinya A. Effect of loading conditions on the fracture toughness of zirconia. J. Prosthodontic Res. 57, 82–87 (2013). 10.1016/j.jpor.2013.01.005 23498598

[b31] Masheder B., Urata C. & Hozumi A. Transparent and hard zirconia-based hybrid coatings with excellent dynamic/thermoresponsive oleophobicity, thermal durability, and hydrolytic stability. ACS Appl. Mater. Interfaces 5, 7899–7905 (2013). 23848181 10.1021/am401992h

[b32] Barriet D. & Lee T. R. Fluorinated self-assembled monolayers: composition, structure and interfacial properties. Curr. Opin. Colloid Interface Sci. 8, 236–242 (2003).

[b33] Tsibouklis J. & Nevell T. G. Ultra-low surface energy polymers: the molecular design requirements. Adv. Mater. 15, 647–650 (2003).

[b34] De G., Chatterjee A. & Ganguli D. Zirconia fibres from the zirconium n-propoxide-acetylacetone-water-isopropanol system. J. Mater. Sci. Lett. 9, 845–846 (1990).

[b35] Spijksma G. I., Bouwmeester H. J. M., Blank D. H. A. & Kessler V. G. Stabilization and destabilization of zirconium propoxide precursors by acetylacetone. Chem. Commun. 1874–1875 (2004). 10.1039/b406012a 15306927

[b36] Fu C. J. *et al.* Influence of Zr/Si molar ratio on structure, morphology and corrosion resistant of organosilane coatings doped with zirconium (IV) n-propoxide. Int. J. Electrochem. Sci. 9, 2603–2619 (2014).

[b37] Elvira M. R. *et al.* Study and characterization of organically modified silica-zirconia anti-graffiti coatings obtained by sol-gel. J. Chem. Chem. Eng. 7, 120–131 (2013).

[b38] Kim C. Y., Ko H. J., Kim D. S. & Choi C. K. Effect of annealing on the bonding structure and dielectric properties of a-C: F thin films. J. Surf. Anal. 12, 218–222 (2005).

[b39] Muralidhara K. S. & Sreenivasan S. Thermal degradation kinetic data of polyester, cotton and polyester-cotton blended textile material. World Appl. Sci. J. 11, 184–189 (2010).

[b40] Zhou X. *et al.* Robust and durable superhydrophobic cotton fabrics for oil/water separation. ACS Appl. Mater. Interfaces 5, 7208–7214 (2013). 23823678 10.1021/am4015346

[b41] Zhang J., Li B., Wu L. & Wang A. Facile preparation of durable and robust superhydrophobic textiles by dip coating in nanocomposite solution of organosilanes. Chem. Commun. 49, 11509–11511 (2013). 10.1039/c3cc43238f 23884359

[b42] Zhu X. *et al.* Robust superhydrophobic surfaces with mechanical durability and easy repairability. J. Mater. Chem. 21, 15793–15797 (2011).

[b43] Zhang Y., Ge D. & Yang S. Spray-coating of superhydrophobic aluminium alloys with enhanced mechanical robustness. J. Colloid. Inter. Sci. 423, 101–107 (2014). 10.1016/j.jcis.2014.02.024 24703674

[b44] Gong M. *et al.* Superhydrophobicity of hierarchical ZnO nanowire coatings. J. Mater. Chem. A 2, 6180–6184 (2014).

[b45] Xue C.-H. *et al.* Self-roughened superhydrophobic coatings for continuous oil-water separation. J. Mater. Chem. A. 3, 10248–10253 (2015).

