# Supplementary material for: Zirconia based superhydrophobic coatings on cotton fabrics exhibiting excellent durability for versatile use
Source: Sci Rep. 2015 Dec 18;5:18503. doi: 10.1038/srep18503 (PMC4683463; doi:10.1038/srep18503)
Supplement: Supplementary Information [file srep18503-s1.pdf]

Supplementary Information

Zirconia based superhydrophobic coatings on cotton fabrics exhibiting excellent durability for versatile use

Indranee Das and Goutam De*

Nano-Structured Materials Division, CSIR-Central Glass and Ceramic Research Institute, Kolkata 700032, India

*Corresponding author

Goutam De

Tel.: +91-33-23223403; Fax: +91-33-24730957; E-mail address: gde@cgcri.res.in

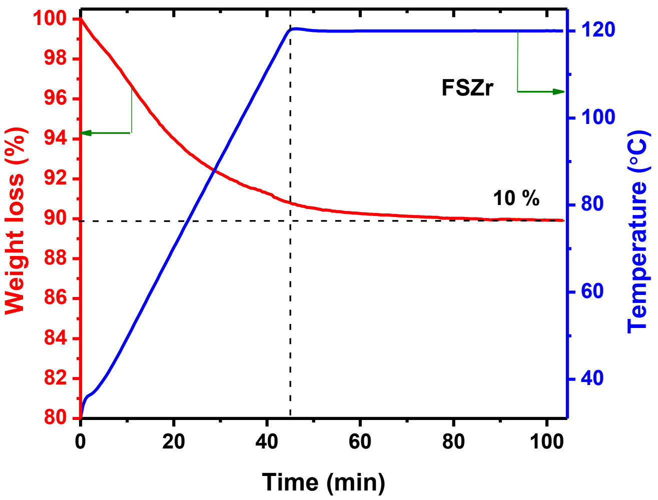

Supplementary Figure S1 Thermogravimetric analysis. TGA of fsZr coating material under 1 h isothermal condition at 120 °C. The sample was previously dried at 60 °C and measurement was performed at a heating rate of 2 °C min^{-1} in static air.

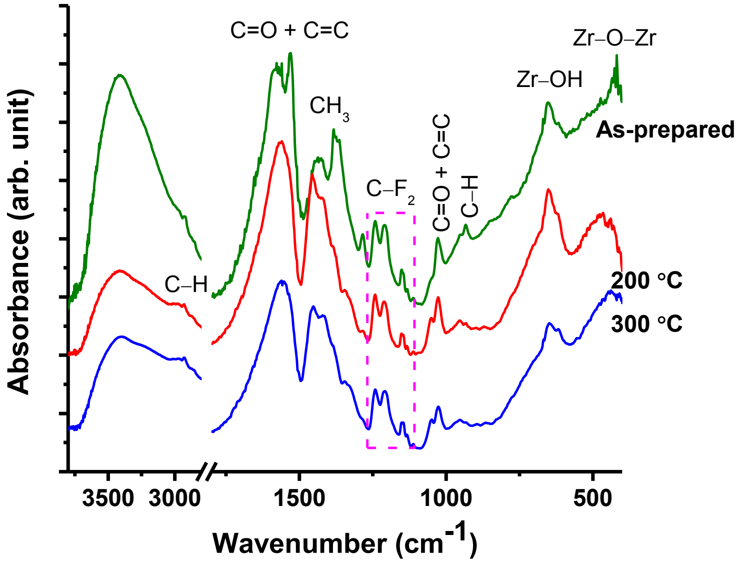

Supplementary Figure S2 FTIR spectra. FTIR spectra of as prepared and heat-treated (200 and 300 °C) fsZr coating materials. The $–CF_2$ groups of the fsZr remained unaffected even after heat-treatment at 300 °C for 1 h in air.

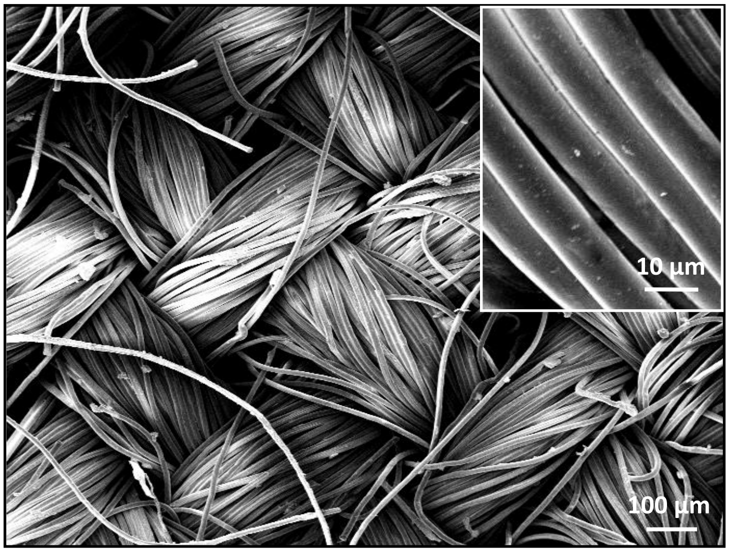

Supplementary Figure S3 FESEM image. FESEM image of the uncoated cotton fabric reveals its tightly woven and smooth surface. Inset shows the higher magnification image.

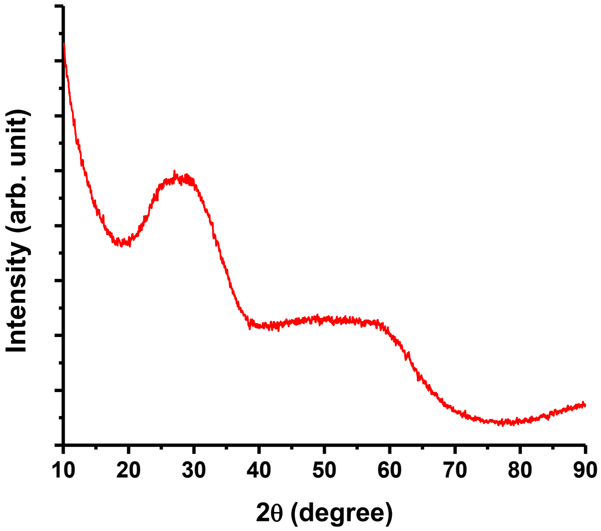

Supplementary Figure S4 Wide angle XRD pattern. XRD pattern shows the characteristic amorphous nature of fsZr coating material heat-treated at 120 °C for 1h.

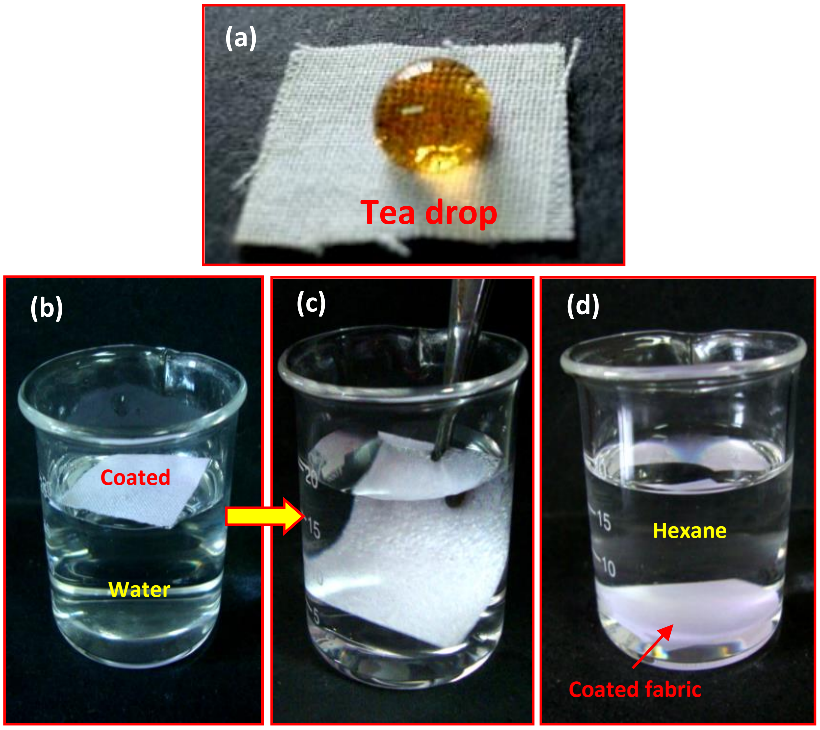

Supplementary Figure S5 Images of fsZr coated cotton fabrics. (a) Image of tea drop on coated superhydrophobic fabric. (b) Image of superhydrophobic fabric floating on the water surface. (c) Coated fabric immersed in water by an external force. (d) Coated fabric went under the beaker containing hexane by free of force showing its superoleophilic nature.

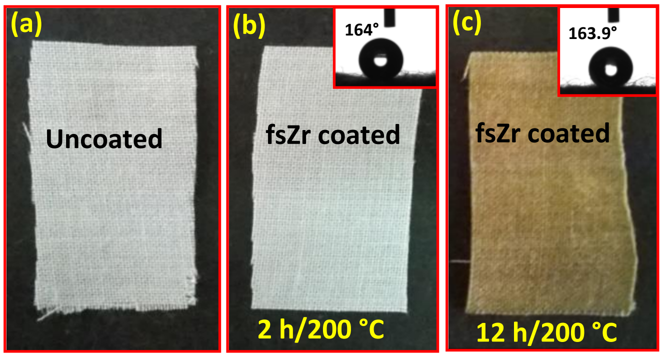

Supplementary Figure S6 Images of coated and uncoated cotton fabrics. Images of (a) uncoated cotton fabric as control without any heat-treatment, (b) and (c) fsZr coated cotton fabrics heat-treated at 200 °C for 2 and 12 h in air, respectively. The change of color in the latter case (c) is due to the partial degradation of cellulose component in cotton. Insets in (b) and (c) show no deterioration of superhydrophobicity of the coated fabrics due to such heat-treatments.

Supplementary Table S1 Changes in water contact angle (WCA) values of coated fabrics after immersion in different organic solvents for 14 d.

**Organic solvent**	**WCA of fabric after immersion (°)**		
	**3 d**	**7 d**	**14 d**
Hexane	162.5±1	161.8±2	160.5±1
DMF	163±1	162.5±1	161±1
Ethylacetate	162±1	161±2	160±2
Ethanol	160±2	159.6±1	158.5±2
Acetone	160.9±2	159.5±2	158.5±1

Supplementary Table S2 Changes in water contact angle (WCA) values and separation efficiency of fsZr coated superhydrophobic fabric before and after 10 cycles of separation.

**Sample**	**Initial contact angle (°)**	**After 10 cycles contact angle (°)**	**Separation efficiency (wt%)**
fsZr coated fabric	163± 1 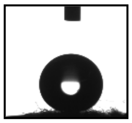	162± 1 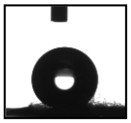	>98

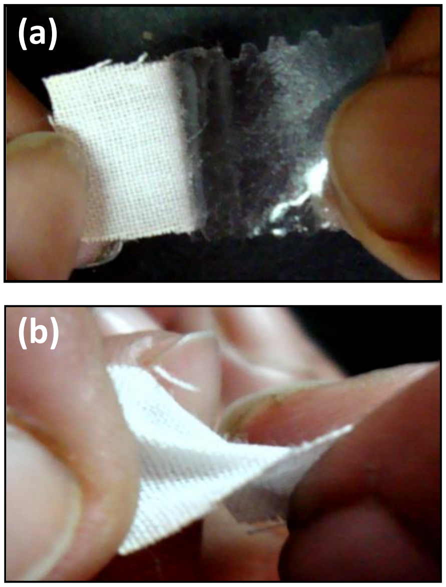

Supplementary Figure S7 Qualitative mechanical stability tests. Qualitative abrasion tests of the fsZr coated superhydrophobic fabric through adhesion of single sided tape (a) and twisting by hand (b).
